# Induced Overexpression of THAP11 in Human Fibroblast Cells Enhances Expression of Key Pluripotency Genes

**DOI:** 10.31661/gmj.v8i0.1308

**Published:** 2019-08-10

**Authors:** Saeid Ziaei, Mostafa Rezaei-Tavirani, Abdolreza Ardeshirylajimi, Ehsan Arefian, Masoud Soleimani

**Affiliations:** ^1^Department of Basic Sciences, Faculty of Paramedical Sciences, Shahid Beheshti University of Medical Sciences, Tehran, Iran; ^2^Student Research Committee, Faculty of Paramedical Sciences, Shahid Beheshti University of Medical Sciences, Tehran, Iran; ^3^Stem Cell Technology Research Center, Tehran, Iran; ^4^Proteomics Research Center, Faculty of Paramedical Sciences, Shahid Beheshti University of Medical Sciences, Tehran, Iran; ^5^Department of Tissue Engineering and Applied Cell Sciences, School of Advanced Technologies in Medicine, Shahid Beheshti University of Medical Sciences, Tehran, Iran; ^6^Department of Microbiology, School of Biology, College of Science, University of Tehran, Tehran, Iran

**Keywords:** Cell Differentiation, Stem Cells, THAP11 Protein

## Abstract

**Background:**

THAP11 is a recently discovered pluripotency factor and described as an important gene that involved in embryonic stem cells self-renewal and embryo development, which works independently with other known pluripotency factors. We aimed to overexpressed the THAP11 gene in primary fibroblast cells to determine the effects of the THAP11 on these cells.

**Materials and Methods:**

The THAP11 gene was amplified using PCR followed by ligation into pCDH vector and lentiviral particle production in HEK293T cells by using psPAX2 and pMD2.G helper vectors. The human fibroblast cells were transduced using viral particles and after confirmation overexpression, the key pluripotency factors were estimated using real-time PCR and changes in proliferation rate was measured by the 3-[4, 5-dimethylthiazol-2-yl]-2, 5 diphenyltetrazolium bromide (MTT) test.

**Results:**

The overexpression of THAP11 in fibroblast cells leads to increase the expression level of *Sox2*, *Oct4*, *Nanog* and *Klf4* as key pluripotency genes and a decrease in proliferation rate according to MTT results.

**Conclusion:**

Our results confirm that we are faced with a molecule with double features, which could be involved in pluripotency and proliferation suppressor simultaneously. It seems that the roles of THAP11 in pluripotency are so complex and attributed to other regulatory molecules.

## Introduction

The ability of the cells to differentiate into other cell types called stemness and the most potent stem cells are pluripotent or embryonic stem cells, which have the most differentiation capability and they can be converted to all types of cells present at the body [1]. Embryonic stem cells (ESCs) are present in the inner cell mass of embryos, and they are producing all the cells of the body except zygote [2]. The proliferation and versatile differentiation potential of the pluripotent stem cells could provide an unlimited source of the cells for therapeutic purposes [3]. As the human ESCs are not accessible easily, we could not achieve the ESCs which a person made from [4]. The scientists are working on the conversion of the differentiated cells into pluripotent stem cells, and they efforts lead to the creation of embryonic-like cells called induced pluripotent stem cells (iPSCs) [5, 6]. Working with ESCs or conversion of somatic cells to iPSCs and using them for therapeutic purposes is not possible without knowing the pluripotency regulation and maintenance factors and pathways. Nowadays, many factors and pathways are reported, which are involved in embryonic state maintenance and regulation, but there is a lot of dark spots in pluripotency regulation factors. The famous key pluripotency genes, which are known as pluripotency markers and are using in the production of iPSCs are *Oct4*, *Klf4*, and *Sox2* [7, 8]. THAP11, as the most recently described member of the THAP domain family, has recently been shown to involved in cell proliferation and described as key pluripotency genes [9]. Knocking out of the mouse THAP11 gene ‒which is named *Ronin* ‒was lethal in early embryonic development stages, while overexpression of *Ronin* in ESCs prevented the cell from differentiation and promoted teratocarcinoma formation [9]. Dejosez *et al*. showed that *Ronin* expression in the adult tissue was negligible [9]. However, Zhu *et al*. believed that the THAP11 gene normally expressed in adult tissue [10]. Also, there are some reports about different expression levels of THAP11 in tumors and various cell types [11-13]. In the molecular level, THAP11 contains an evolutionarily conserved zinc finger motif called THAP Domain, which has sequence-specific DNA-binding activity [14]. The proposed mechanisms of *Ronin* action in pluripotency is the regulation of the cell cycle controller genes by binding to a hyperconserved shared enhancer including *HCF1* and *ZNF143* [15]. Despite several reports about the THAP11 action in the stem cells, the exact mechanisms of THAP11 in pluripotency still unclear and the most researches were done on ESCs. Since the expression level of THAP11 in differentiated cells is negligible, we aimed to changes the expression level of THAP11 by genetic engineering in fibroblast cells for estimating the effects of THAP11 on pluripotency factors in differentiated cells.

## Materials and Methods

### Molecular Cloning of THAP11

The lentiviral CDH-CMV-MCS-EF1-GFP-T2A-Puro vector (System Biosciences, California, USA), which harbors Amp and Puro (ampicillin and puromycin resistance genes) as selective markers in prokaryote and eukaryote hosts respectively and GFP as a reporter in eukaryote hosts was used for this study. Genomic DNA was extracted from human blood using QIAamp DNA Blood Midi Kit (Qiagen, Hilden, Germany) according to the manufacturers’ protocol. The THAP11 gene was obtained using polymerase chain reaction (PCR) amplification by primers described in Table-1 with Phusion DNA polymerase (New England Biolabs, Beverly, MA, USA) and standard reaction mixture by temperature settings according to Table-2. The PCR product was digested with XbaI/EcoRI and ligated into the digested vector fragment by T4 DNA ligase (Thermo Fisher Scientific, Rockford, IL, USA).

### Lentiviral Production and Transduction

The lentiviral transduction method was used for introduction of THAP11 gene into fibroblast cells. To produce viral particles, HEK293T cells were co-transfected with pCDH-THAP11 and psPAX2 and pMD2.G using the Lipofectamine 3000 reagent (Thermo Fisher Scientific, Rockford, IL, USA), according to the manufacturers’ application note. Harvested supernatant filtered through a 0.45 μm filter and used as rich viral source for transduction. The fibroblast cells were seeded in T25 cell culture flask until they reach a cell density of approximately 70-80% and then 500 µl of filtrated virus mixed with 40 mg polybrene was added to the flask and incubated for 24 h followed by replacement of the culture medium. Afterward, 48h after transduction, the cells were examined by fluorescence microscopy for estimation of the transfection efficiency.

### Cell Proliferation Assessment

Normal and recombinant cells were plated in 96-well microplates at a cell density of 3 × 10^3^ cells/well. Cell proliferation was detected every 24 h by adding 5 mg/ml 3-[4, 5-dimethylthiazol-2-yl]-2, 5 diphenyltetrazolium bromide (MTT) solution followed by 3h incubation. Formazan crystals were solubilized 150 μl dimethyl sulfoxide for 30 min. Optical density (OD) was recorded on a microplate reader at 570 nm[16].

### RNA Extraction, cDNA Synthesis, and Quantitative PCR Analysis

In order to analyze the effect of THAP11 overexpression on key pluripotency genes, quantitative PCR was performed to assay *Oct4*, *Sox2*, *Klf4*, and *Nanog* genes for 72h after transduction. RNA extraction was done by NucleoSpin® RNA kit (Macherey-Nagel Gmbh & Co., Düren, Germany) according to the manufacturer’s protocol. Also, the DNAse enzyme was used for elimination of residual DNA content of final extracted RNA as described in the kit protocol. cDNA synthesis was performed with RevertAid Reverse Transcriptase (Thermo Fisher Scientific, Rockford, IL, USA) using Random Hexamer Primer according to Standard protocol. The quantitative PCR was carried out with SYBR Green PCR Master Mix (Thermo Fisher Scientific, Rockford, IL, USA) by applied bioscience StepOnePlus Real-Time PCR System according to the standard protocol with primers described in Table-3.

### Data Analysis

Real-time PCR data analysis was performed using REST2009 software (Qiagen, Germany) [17], and subsequent data was analyzed using Microsoft Excel 2010, and student’s t-test. A P-value of less than 0.05 was considered as statistically significant [18].

## Results

**Figure 1:**
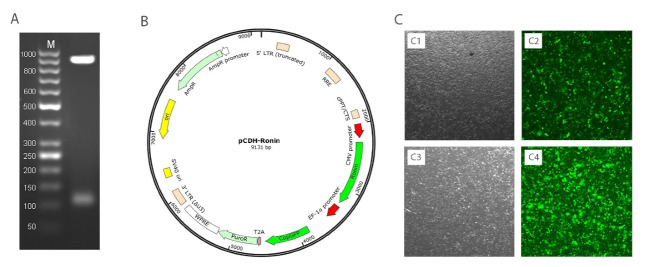
THAP11 is containing lentiviral particle production. **A:** Agarose gel electrophoresis results of PCR amplification of THAP11 Gene; **B:** The final recombinant construct resulted from the cloning of THAP11 gene into the pCDH vector; **C:** Expression of GFP in HEK293T cells after transfection of lentiviral packaging system under fluorescence microscopy (×100). **C1:** Control cells (empty pCDH vector) in optical microscopy; **C2:** Control cells in fluorescent microscopy; **C3:** pCDH-THAP11 containing cells in optical microscopy; **C4:** pCDH-THAP11 containing cells in fluorescent microscopy.

**Figure 2:**
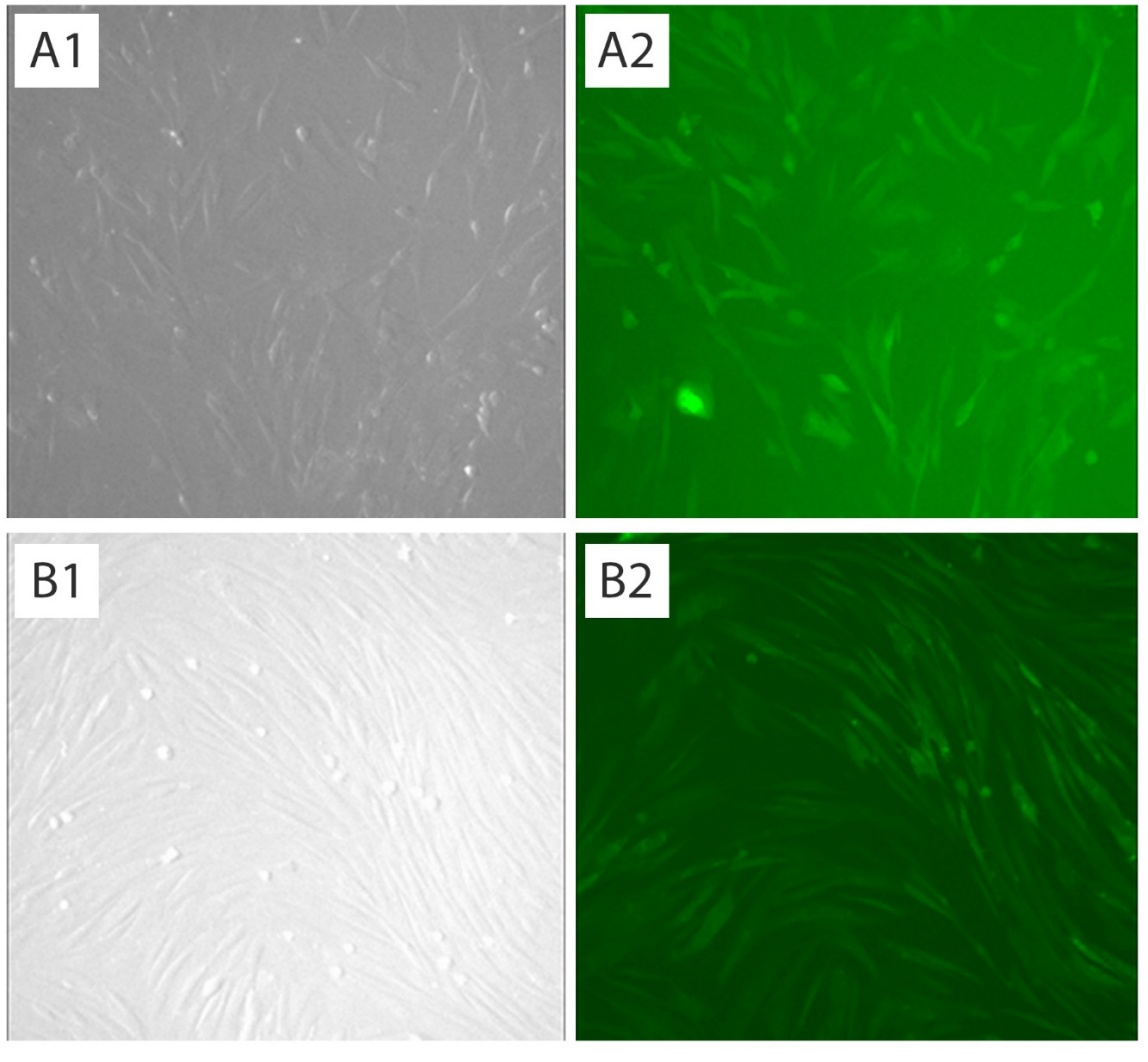
Expression of GFP in human fibroblast cells, 48h after transduction with lentiviral particles in fluorescence microscopy (×100). **A:** pCDH-THAP11 is containing cells; **A1:** Optical microscopy, **A2:** Fluorescent microscopy; **B:** Control cells (empty pCDH vector); **B1:** Optical microscopy, **B2:** Fluorescent microscopy.

**Figure 3:**
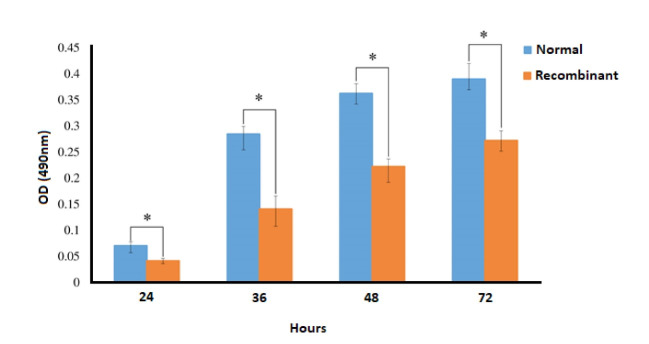
The proliferation test of the THAP11 overexpressed fibroblast cells (recombinant cells) and normal cells, ^*^ P<0.05

**Figure 4:**
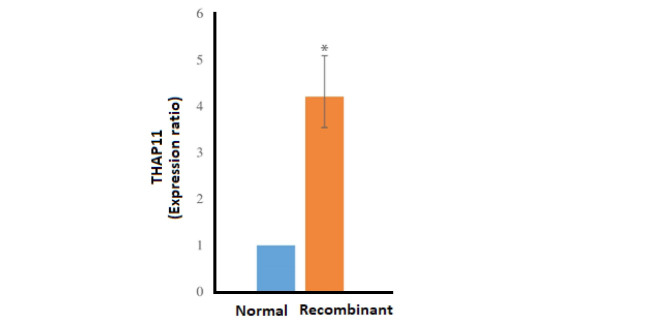
Quantitative real-time PCR analysis of the expression ratio of THAP11 gene in recombinant cells and the control, ^*^P<0.05

**Figure 5:**
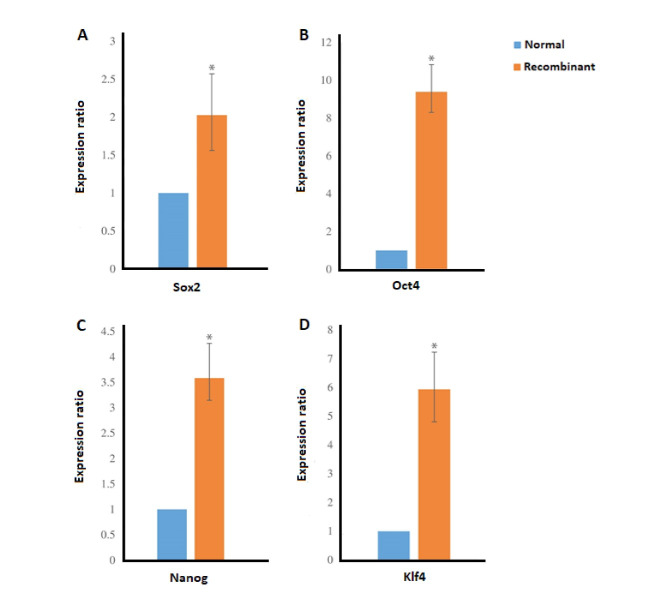
Quantitative real-time PCR analysis of the expression of Sox*2*(**A**), *Oct4*(**B**), *Nanog* (**C**) and *Klf4* (**D**) genes in THAP11 overexpressed cells and the control, ^*^P<0.05

The PCR results of THAP11 gene in agarose gel electrophoresis is shown in Figure-1A and the schema of the final constructed pCDH vector, which is named pCDH-THAP11, is shown in Figure-1B. The lentiviral production was done according to described method with approximately 70% transfection efficiency according to fluorescence microscopy in 24h post-transfection (Figure-1C). The cultured fibroblast cells were exposed to harvested viral particles for 24h, and the fluorescent microscopy results are represented in Figure-2. MTT results show that the growth rate of the fibroblast cells was significantly slower than the control group and the proliferation rate decreased by 37% after 72h post-transfection (Figure-3). The real-time PCR method was used to confirm the overexpression of THAP11 in recombinant cells and result showed that the expression of THAP11 gene increased 4.2 fold in comparison with the control cells (Figure-4). Although real-time PCR analysis of pluripotency genes showed that the *Oct4*, *Sox2*, *Klf4,* and *Nanog* expression were increased 9.3, 2, 5.9, and 3.6 folds, respectively. Increasing the expression of core pluripotency factors reveals the role of THAP11 gene in the pluripotency regulation (Figure-5).

## Discussion

The maintenance of a pluripotency state in ESCs is a complex mechanism, which depends on various regulatory factors [19]. Among those, the *Ronin* gene is one of the latest introduced ones reported by Dejosez *et al*. (2008)[20]. The THAP11 is a DNA binding protein which has potential to influence diverse cellular activities such as differentiation [21, 22], proliferation [13, 23], and cell migration [12, 24]. Also, there are several reports about the roles of THAP11 in multiple cell regulatory mechanisms and diseases. In this study, the THAP11 gene was overexpressed in primary fibroblast cells, and subsequent analysis has shown that the THAP11 overexpression had negative influences on the proliferation rate and positive effects on the expression of key pluripotency genes. MTT results demonstrated that the overexpression of THAP11 decreased the proliferation rate of human fibroblast cells by about 40 % and it compatible with Zhu *et al*. [25] and Nakamura *et al*. [13] reports. Zhu *et al.* [25] represented that the THAP11 are ubiquitously expressed in normal tissues and its overexpression could prevent the cell growth by *c-Myc* suppression. Nakamura *et al.* [13] reported that the overexpression of THAP11 inhibited chronic myeloid leukemia cell proliferation by suppression of *c-Myc*. The gene expression analysis shows that the overexpression of THAP11 had positive effects on expression levels of key pluripotency genes. It is proved that the human THAP11 is involved in pluripotency regulation. In 2008, Dejosez *et al*. reported that the mouse THAP11 serves as a key regulator of pluripotency [9]. However, Zhu *et al*. proposed that the human THAP11 is different from mouse THAP11 – which is named *Ronin* by Dejosez *et al*. – and it is not involved in pluripotency [25]. Our results show that in spite of the cell growth inhibitory effects of THAP11, which is proved by the MTT test, it involves in pluripotency with a positive regulatory role.

## Conclusion

According to our results, we believe that although THAP11 is involved in pluripotency, we are facing with very complex regulation network that could not be interpreted simply only by one gene investigation, and it should be investigated alongside with the other members of the regulatory networks. Further comprehensive studies are required to clarify these contradictions.

## Acknowledgment

I want to show my warm thanks to all the people that I have worked with during these years in Stem Cell Technology Research Center.

## Conflict of Interest

Authors declare there is no any conflict of interest.

**Table 1:** Cloning Primer Sequences.

	**Extra Sequences (5’->3’)** ^*^	**Binding Sequence (5’->3’)** ^**^	**Length**	**Tm** ^***^
**Forward primer**	F: GCTCTAGAG	F: CATGCCTGGCTTTACGTGC	9 + 19	59.86
**Reverse primer**	R: CGAATTCC	R: TCACATTCCGTGCTTCTTG	8 + 19	55.85

^*^ Restriction enzyme binding site ^**^ Binding sites of the primers ^***^ Calculation of the Tm is based on binding part of the primers

**Table 2:** Cycling Conditions for a Cloning PCR Reaction

**Steps**	**Cycles**	**Temperature**	**Time** **(seconds)**
**Initial denaturation**	1	98°C	30
**Denaturation**	30	98°C	10
**Annealing**	30	60°C	30
**Extension**	30	72°C	30
**mFinal Extension**	1	72°C	600
**Hold**	1	4°C	ꚙ

**Table 3:** Real-Time PCR Primer Sequences.

**Gene name**	**Sequence (5’->3’)**	**Length**	**Tm**
**THAP11**	F: CTTGTCGTCAGGCACCAC	18	58.36
	R: CATCTCTTTCATCTTCACTTCC	22	54.57
***Sox2***	F: GGACTGAGAGAAAGAAGAGGAG	22	57.28
	R: GAAAATCAGGCGAAGAATAAT	21	52.29
***Oct4***	F: GAAGCTGGAGAAGGAGAAGCTGG	23	62.8
	R: CAAGGGCCGCAGCTTACACAT	21	63.58
***Nanog***	F: CTCCTTCCATGGATCTGCTTATTC	24	59
	R: AGGTCTTCACCTGTTTGTAGCTGAG	25	62.48
***Klf4***	F: CCCAATTACCCATCCTTCC	19	54.87
	R: GTGCCTGGTCAGTTCATC	18	55.36
